# High-speed camera system for efficient monitoring of invasive plant species along roadways

**DOI:** 10.12688/f1000research.141992.2

**Published:** 2024-10-04

**Authors:** Mads Dyrmann, Søren Kelstrup Skovsen, Peter Hviid Christiansen, Mikkel Fly Kragh, Anders Krogh Mortensen

**Affiliations:** 1 The AI Lab ApS, Aarhus, 8210, Denmark

**Keywords:** high-speed camera, invasive plant species, roadside monitoring

## Abstract

Invasive plant species pose ecological threats to native ecosystems, particularly in areas adjacent to roadways, considering that roadways represent lengthy corridors through which invasive species can propagate. Traditional manual survey methods for monitoring invasive plants are labor-intensive and limited in coverage. This paper introduces a high-speed camera system, named CamAlien, designed to be mounted on vehicles for efficient invasive plant species monitoring along roadways. The camera system captures high-quality images at rapid intervals, to monitor the full roadside when following traffic speed. The system utilizes a global shutter sensor to reduce distortion and geotagging for precise localistion. The camera system makes it possible to collect extensive data sets, which can be used for a digital library of the invasive species and their locations, but also subsequent training of machine learning algorithms for automated species recognition.

## Introduction

Invasive plant species have emerged as a significant ecological concern due to their adverse impacts on native ecosystems ( Gentili et al., 2021). Roadways and their adjacent areas provide a corridor for the rapid spread of invasive plants ( Okimura et al., 2016; Lembrechts et al., 2014). Consequently, the development of effective monitoring tools and methodologies is essential for identifying and managing these invasive species, thereby mitigating their ecological consequences.

Traditionally, identifying and mapping invasive plants relied on manual observations and record-keeping. However, conducting thorough surveillance poses challenges due to expansive road networks and vast land areas. To effectively spot these invasive plants, a trained individual must be transported at a pace that allows the person to recognizing them, a task often unattainable when driving at normal speeds on highways. Additionally, due to the varying flowering times among different species, revisiting the same areas multiple times throughout a growing season becomes a necessity to capture the variation within each species. For example, we do not expect an AI model trained to recognize lupins ( *Lupinus polyphyllus*) in bloom to be able to recognize them when wilted.

In order to address the challenges associated with intensive monitoring of roadside areas and enhance the effectiveness of monitoring invasive plant species along roadways, we propose a high-speed camera specifically designed for mounting on vehicles. By utilizing the existing infrastructure of vehicles traversing road networks, such a camera can maximize the spatial coverage and minimize resource requirements, making invasive plant monitoring more cost-effective and time-efficient.

Other studies have focused on automatic monitoring of invasive plant species and investigation of ecological change. Pinzani and Ceschin (2023) engage in monitoring invasive plants along the road network. For this, they use smartphone cameras, as a cheap means of collecting images with geolocation. In their study, they focus on three invasive species in the Tuscany region (central Italy) and they conclude that “Reproducibility, accessibility, and expeditiousness make the SPM [smartphone-monitoring, ed.] method easily exportable, widely usable, and adaptable to different environmental contexts and geographical areas” ( Pinzani and Ceschin, 2023, p. 8). Likewise, Surya (2023) demonstrates that a smartphone camera mounted on a bike, can be used for monitoring knotweed plants from a bicycle. Depauw et al. (2022) review the use of ground-based photos in biodiversity and community ecology, phenology, global change ecology and landscape ecology. They find that photography can fundamentally contribute to the understanding of ecosystem responses and that the use of time-lapse cameras may provide an alternative for time consuming observational field studies. However, the right camera technology is not enough, as investments must also be made in proper data handling and analysis.

This paper presents the design, capabilities, and performance of the proposed high-speed camera equipment. We emphasize the importance of capturing high-quality images while maintaining the necessary speed for effective monitoring. The proposed camera design aims to enhance the frequency and scalability of invasive plant monitoring, providing valuable insights for ecologists engaged in ecological restoration and invasive species management programs. The camera system, named CamAlien, is presently undergoing testing in 11 countries across Europe and its surrounding regions.

The subsequent sections of this paper delve into the technical aspects of the camera design, including its hardware specifications, software, and mounting on vehicles. We also present sample images from field trials conducted along selected road sections, demonstrating the image quality of the camera system when used at high speed for invasive alien plant species detection. Finally, we discuss the future directions of this technology in the context of invasive alien species monitoring.

## Camera system overview

**Figure 1.**
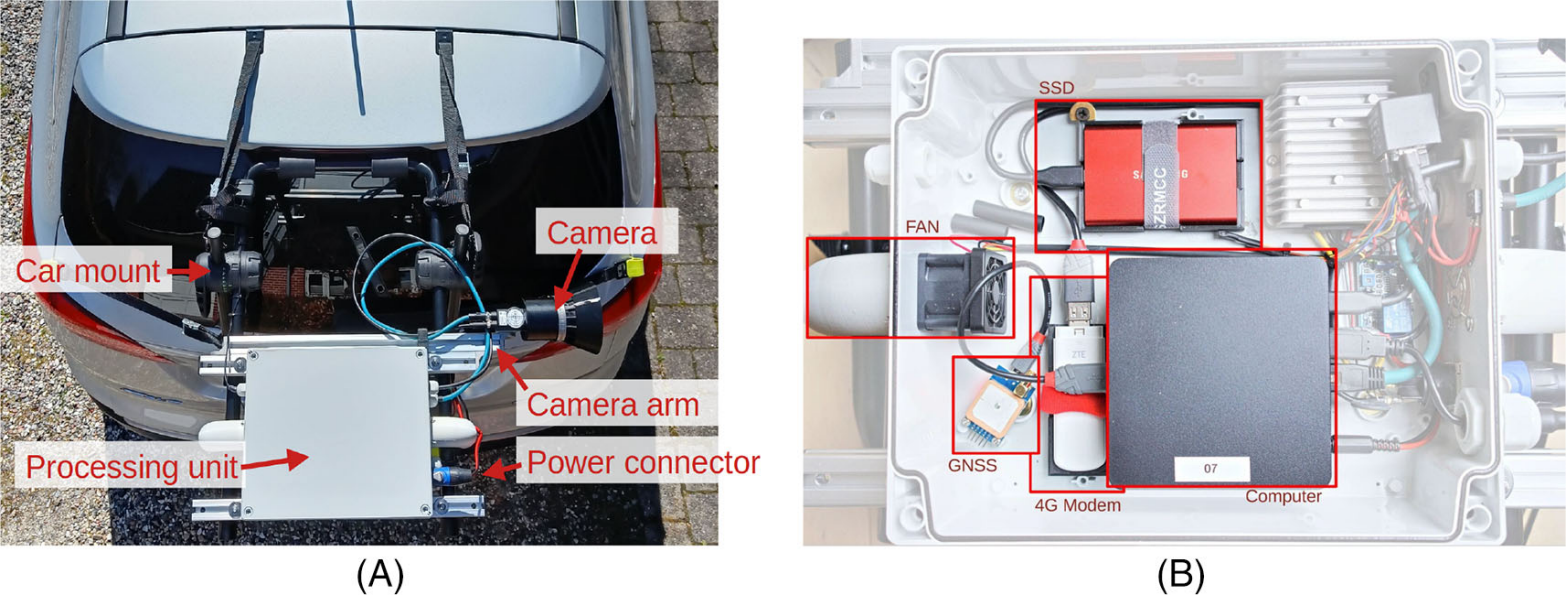
A) Overview of camera system that is mounted on the boot lid of a car. B) Internal of processing unit, which consists of computing device, GNSS, modem, power supply, and storage.

The following sections provide an overview of the camera hardware and software. Figure 1 shows the camera system mounted on the boot lid of a car using a universal mount. The camera system is enclosed and rainproof, but it is recommended to take pictures only in dry weather, as diffraction from rain on the lens can significantly reduce the image quality.

### Camera module

One of the main components of the system is the camera module itself. An important consideration in designing the camera module is the image sensor. The image sensor must be sensitive enough to allow for a short shutter time, minimizing motion blur when driving at high speeds. If the shutter speed is too long, it effectively reduces the horizontal resolution in the image, making object recognition more challenging. Another factor in selecting a camera sensor is whether to use a rolling or global shutter sensor. Traditional rolling shutter sensors, commonly found in consumer cameras, can introduce distortions in fast-moving scenes by sequentially capturing different parts of the image frame. In contrast, a global shutter sensor captures the entire image simultaneously, eliminating rolling shutter artifacts and ensuring accurate scene rendering. This feature is crucial when capturing images at high speeds, such as when monitoring invasive alien plant species along roads from a moving vehicle, as it maintains image integrity and enables reliable analysis and species identification. We have opted to use a camera module from Lucid Vision Labs, featuring an IMX545 global shutter sensor from Sony Semiconductor. This sensor has a size of 1/1.1" and boasts a resolution of 12.28 megapixels. We chose this resolution based on a previous study that demonstrated the capability of detecting frequently occurring invasive species along Danish motorways using a 12 megapixel sensor and a 46° horizontal field of view ( Dyrmann et al., 2021). Opting for a higher resolution with the same field of view would necessitate a shorter shutter time, potentially leading to increased image noise. Additionally, it would demand a higher bandwidth from the camera to the storage device. Therefore, resolution represents a trade-off between having enough detail for recognizing invasive alien plants and minimizing image noise.

### Recording, coverage and field of view considerations

The lens is important in the design of a camera system, as it influences the image clarity, image distortion, and field of view. The focus distance must be suited to the distance to the plants, and the aperture should be small enough to ensure the *depth of field* covers the width of the roadside, but large enough to illuminate the sensor sufficiently. These plants can establish themselves on the road’s periphery, often left partially untouched, unless the vegetation interferes with traffic safety. The distance from the camera to the vegetation and the car’s speed are vital factors for determining the appropriate focal length, shutter speed, and image sampling rate. The camera can be rotated, but for monitoring the road side, it would typically be pointed perpendicular to the travel direction so that it points directly towards the road side. Therefore, the following estimates are based on this camera orientation. If the camera was mounted with a different orientation, it would have fewer demands on fast exposure. The estimated distance from the camera to the invasive plants at the roadside is 7 meters. Danish motorways typically feature a hard shoulder of around 3 meters and at least 1.5 meters vegetation free space ( Madsen et al., 2018), but often more. Moreover, the distance from the vehicle to the shoulder is approximately 1 meter. In other countries, there are often no continuous shoulders along motorways, making the distance from the vehicle to vegetation shorter. Therefore, the focus distance is set to 5 meters, with a small aperture to achieve a deep depth of focus. The camera system is designed to capture images at a rate of up to 10 frames per second, with each frame having a resolution of 12 megapixels. This frame rate combined with a 12 mm lens (Computar V1226-MPZ) that provides a 50.1° horizontal field of view and 37.8° vertical field of view allows for coverage of the full roadside at a working distance greater than 3.85 meter, when the vehicle is traveling at 130 km/h.

When the camera is mounted 1.5 meters above the ground and is not tilted, and the distance from the camera to the vegetation is 7 meters, the camera can capture vegetation that is up to 3.3 meters in height. The ground sampling distance at 7 meters is 0.6 pixels per millimeter, which we consider sufficient, given that the driver or passenger should also be able to see the plants. The frame rate is limited by the 1-gigabit Ethernet connection from the camera. However, the processing unit has a 2.5-gigabit Ethernet port, which would allow us to add an Ethernet switch and a second camera. That second camera could, for example, capture the opposite roadside or the road surface.

**Figure 2.**
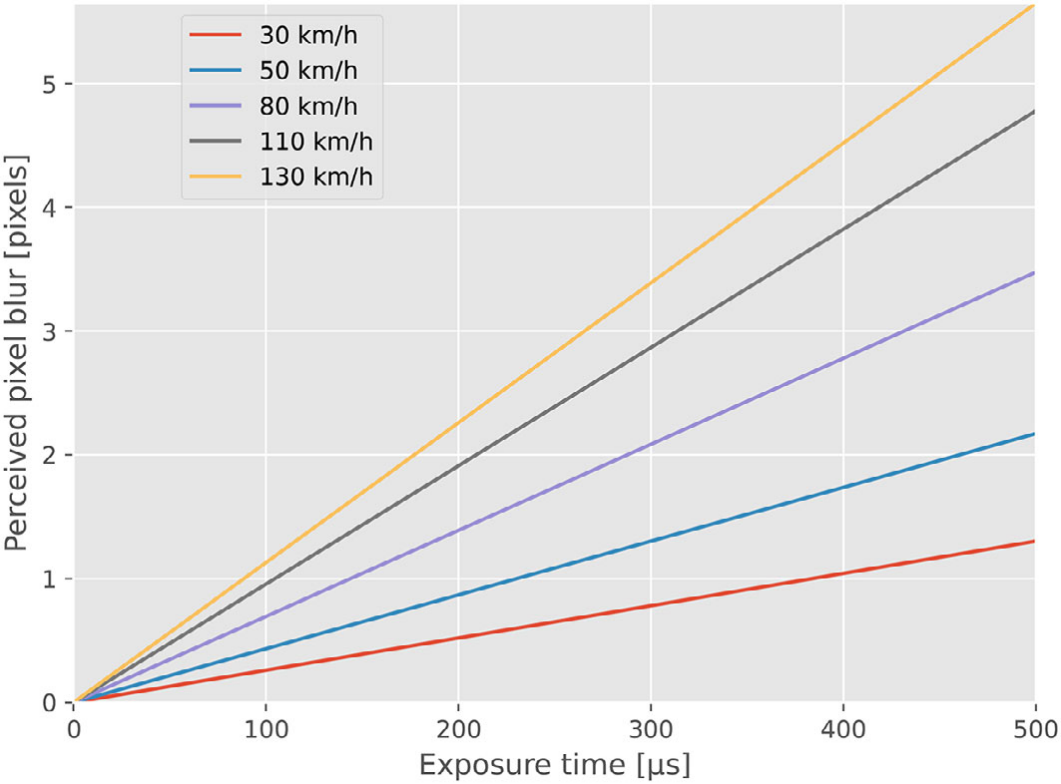
Motion blur when recording objects at a distance of 7 meters. Due to the reliance on natural light, the exposure time in use will depend on the factors such as time of day, weather and season.

The camera needs to function effectively under various lighting conditions since the images solely rely on natural illumination. To ensure uniform lighting across the images, it is essential to continually adapt the camera’s exposure time and gain according to the intensity of sunlight. Consequently, the camera’s settings are configured to prioritize adjusting the exposure time based on the driving speed, so that motion blur is avoided. If additional illumination is still required, the camera will increase the analog gain. It is important to note that both the exposure time and the driving speed impact the sharpness in the images through motion blur, whereas the analog gain adds ISO (International Organization for Standardization) noise. Due to the optical Bayer filter on the camera sensor, the perceived motion blur in the RGB image is approximately half of the theoretical counterpart. Figure 2 illustrates the correlation between exposure time, driving speed, and perceived motion blur, assuming a camera-to-object distance of 7 meters. When driving 130 km/h the maximum perceived blur is less than three pixels, which we consider to be low considering a horizontal resolution of the image of 4096 pixels.

### Remote control

A typical use case for a camera system like this is to build a digital library of detected invasive plant species. Since the camera can capture 10 images per second, it can be an infeasible task to review the images afterwards and identify the invasive plants, whether for direct registration purposes or to create training data for automatic detection.

**Figure 3.**
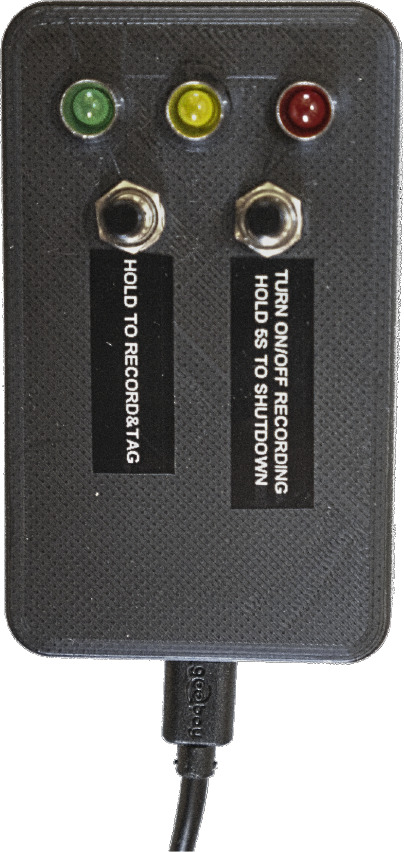
Remote control to tag images, start/stop recordings, and display the system’s status through LEDs.

To facilitate the subsequent annotation of images, we propose a physical remote control shown in Figure 3, that allows marking the locations of invasive plants during recording. When a button on the remote control is pressed, a tag is written into the file name of the images, making it easy to later retrieve the image sequences containing invasive plant species. A time-consuming manual review of the tagged images is still required to annotate invasive plants and build a digital library, but the process has been significantly accelerated. The remote control also enables starting and stopping the recording, checking the system’s status *via* LEDs, and turning off the camera system. To ensure safe driving, the remote control requires the presence of a second passenger in addition to the driver.

### Geotagging images

Accurate spatial information is crucial for effective monitoring and management of invasive plant species. To ensure precise geolocation data, the camera system is equipped with a GNSS (Global Navigation Satellite System) module (U-blox NEO-7M). This module combines localization from GPS (Global Positioning System), GLONASS (Global’naya Navigatsionnaya Sputnikovaya Sistema), and Galileo, to determine the accurate position and time of each image. By geotagging the images, we enable spatial mapping of invasive species distributions and facilitate spatial analysis, aiding in targeted management strategies and accurate assessment of spread patterns. The GNSS module logs the full road stretch traveled, so it is also possible to see where data has been collected but no invasive planets have been observed.

### Power supply and fans

When mounted on the car, the system is powered from the car’s 12 V auxiliary power outlet, *i.e.,* the cigarette lighter socket. Although all of the internal components of the camera system are designed to run at 12 V, the supplied voltage from the car can fluctuate significantly while in operation. During normal operation, the car generator will increase the supply voltage to approximately 13.8 V. The starter motor, however, leads to momentarily voltage drops of 1–2 volts when in use, causing the internal components to turn off if powered directly. To manage these expected fluctuations, as well as vehicles with a 24 V electrical system, a DC-DC converter (Supernight DC310-8 40to12v6a) has been integrated into the camera system. This guarantees a stable 13.8 V internal power supply, provided that the outer supply is in the range of 8–40 V. To keep the system from overheating during summer temperatures, a high static pressure fan (Sanyo Denki 9GV0412P3G03) is mounted on the downfacing intake within the system enclosure. While the downfacing intake and exhaust prevent liquids from entering the system, a replaceable air-filter within the intake keeps out dust and other unwanted materials.

### Modem

To facilitate remote access to the system, the camera has a 4G modem (ZTE MF833U1). When an active SIM card is inserted, the system automatically connects to a remote server when powered on. This allows the system to report online statistics such as road coverage, system health, remaining storage space, *etc.* Moreover, in areas with adequate internet reception, recorded images may be transferred in real time to a centralized server.

### Mount and camera arm

**Figure 4.**
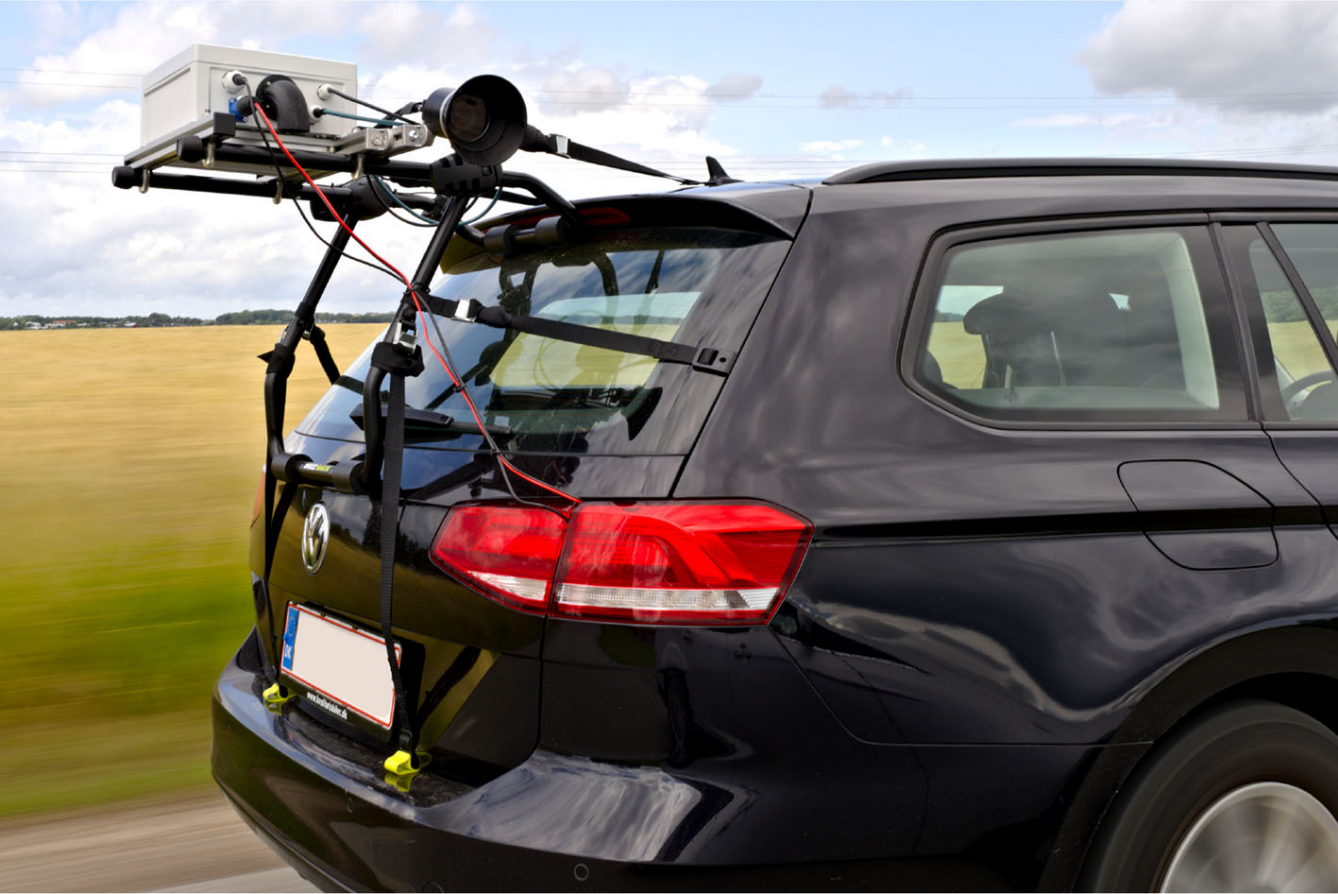
The car-mounted camera system in operation. Two cushioned bars are adjusted and positioned onto the boot lid, where six straps are holding the system in place.

To ensure physical compatibility with a wide range of vehicles, the camera system is designed to be mounted on the boot lid using an adjustable rack (Buzzrack Beetle) as shown in Figure 4. The rack provides a secure and stable platform for attaching the camera equipment, allowing it to be easily installed and removed without causing any damage to the vehicle. The remainder of the system is based on 30×30 mm extruded aluminum frames to ensure a light and durable system. The camera sensor is mounted on a slidable arm that allows the camera sensor to be positioned up to 60 cm closer to the roadside. Using a provided vertical extension arm, the slidable arm can be angled freely up- or downwards, providing vertical adjustments of the camera sensor of up to ±45 cm. To support accurate orientation of the images, the camera sensor is mounted onto the slidable arm with a series of joints that give additional 3 degrees of freedom, corresponding to adjustments of yaw, pitch, and roll. This flexibility enables comprehensive coverage of the road environment across diverse car models, ensuring the same data quality for accurate species identification and monitoring.

### Software

**Figure 5.**
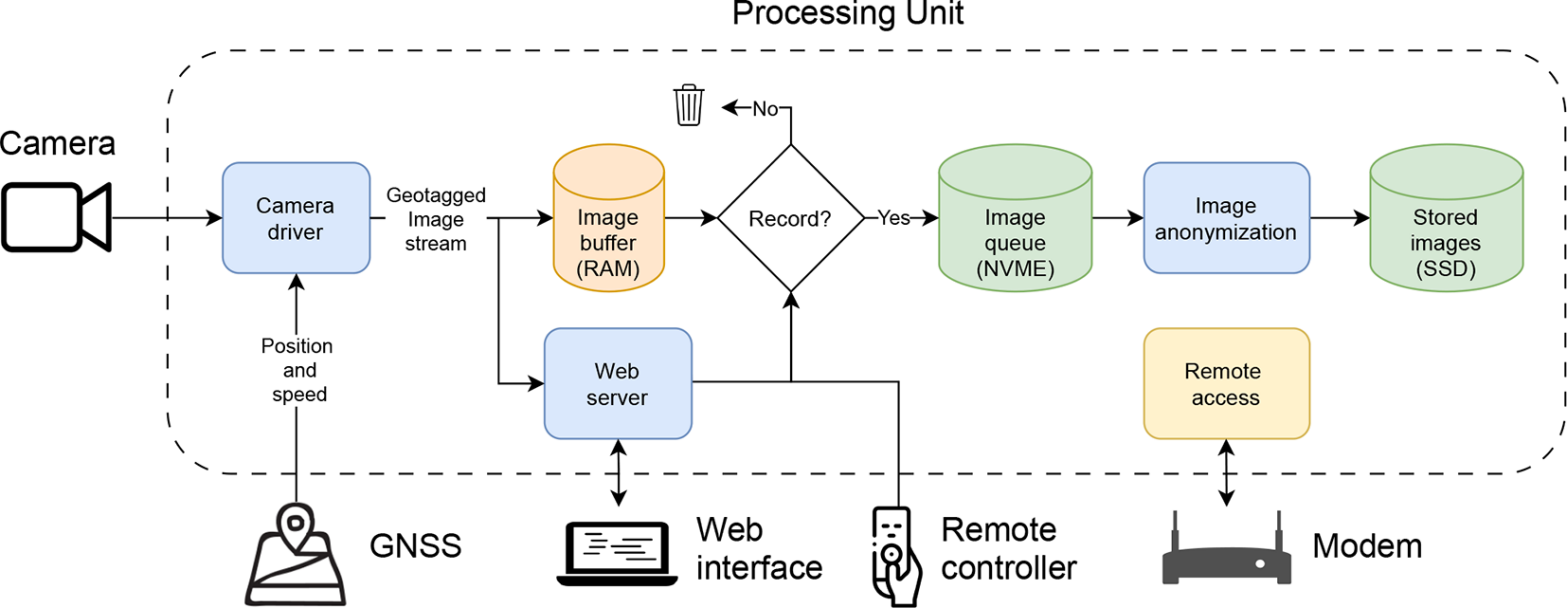
Flowchart illustrating connection and communication between software components.

The processing unit of the system includes an Intel NUC12WSHi5 compute element with the Ubuntu operating system that is connected to the camera, the GNSS module, the modem, and the remote controller. Figure 5 illustrates with a flowchart how different components of the system are connected, and how images flow from the camera, through the memory (RAM) and an internal queue, and are finally stored at an external SSD. The internal queue can hold up to 2 Terabytes corresponding to recording 4.7 hours of raw images at 10 fps *without* off loading to the external ssd, but with an external drive plugged in, this extends to around 10 hours of continuous recording. The communication between components is handled with the Robot Operating System (ROS) ( Stanford Artificial Intelligence Laboratory et al., n.d.). At system startup, the camera driver configures the camera, ensuring that images are taken at the desired frame rate and shutter speed. It then receives and decodes images in real time, adds metadata in terms of timestamp and geotags, and streams the images to the web interface (if a user is connected) and a temporary image buffer. The buffer allows the user to record images for a configured number of seconds back in time, when a button is pressed in either the web interface or with the physical remote controller. When recording is active, raw images are temporarily stored on a 2 terabyte fast internal NVME disk. From here, they are anonymized asynchronously using an AI model (YOLO) that automatically detects and removes people, bikes, and vehicles from the images. Finally, the anonymized images are stored to an external SSD that can be accessed by the user.

### Web interface

**Figure 6.**
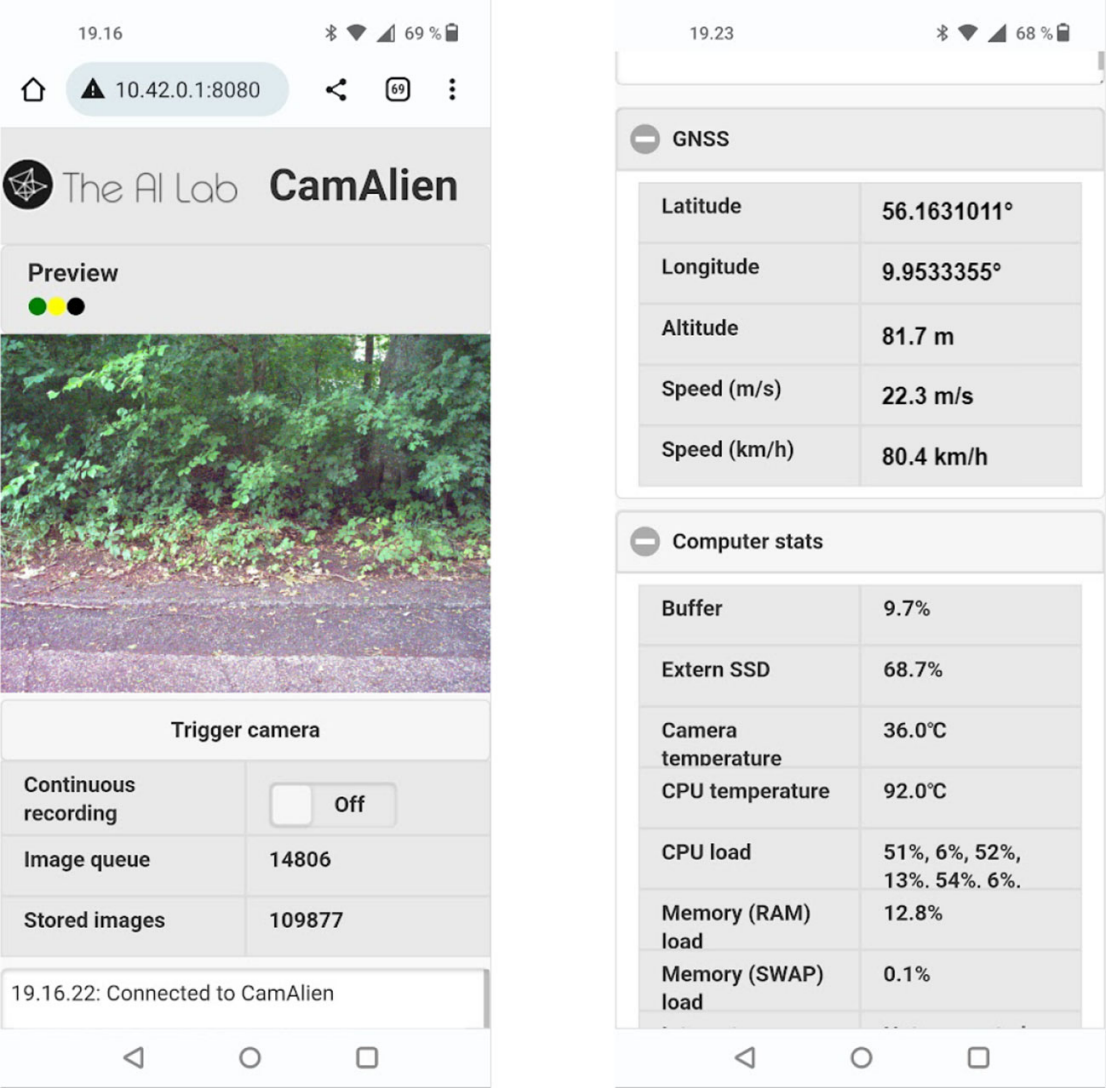
Web interface screenshots. Left: live video stream from the camera system, buttons for triggering the camera and enabling continuous recording, length of image queue and stored images, and status window. Right: GNSS info (position and speed), and system information (disk utilization, temperatures, and CPU and memory load).

While the remote control provides a simple and easy-to-use interface to the camera system, the web interface provides a more complex, flexible and detailed interface to the camera system. The two main features of the web interface are the live video stream from the camera system’s internal image buffer and the status field ( Figure 6, left). The live video stream allows the user to preview the images as real-time feedback of the image content and field of view, allowing the user to quickly adjust the camera position and orientation. The status field provides timestamped human-readable status messages, which elaborates the remote control’s status LEDs, *e.g.* software failure, overheating, and disk utilization. This enables the user to more quickly solve the problem at hand.

Between the two main features, two counters show the number of images in the image queue on the internal disk and the stored images on the external disk ( Figure 6, left), allowing the user to assess whether images are stored while recording, and if the camera system is still off-loading images to the external disk after a long day of recording.

In addition, the web interface displays current GNSS information and computer stats such as disk usage, CPU and camera temperature ( Figure 6, right) and allows the user to trigger the camera, enable continuous recording, and shutdown the camera system.

The web interface was implemented using standard ROS packages and Javascript libraries to ensure compatibility with the rest of the system and easy scalability on the user’s device(s), respectively.

## Sample images

**Figure 7.**
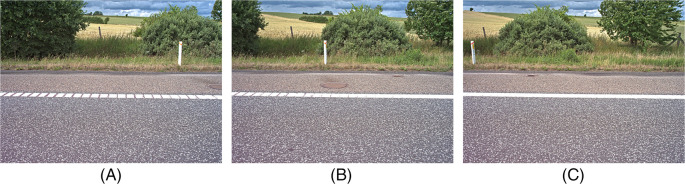
A sequence of images acquired when driving 107 km/h. The images overlap, which ensures full coverage of the roadside and it is possible to recognize the plant species.

**Figure 8.**
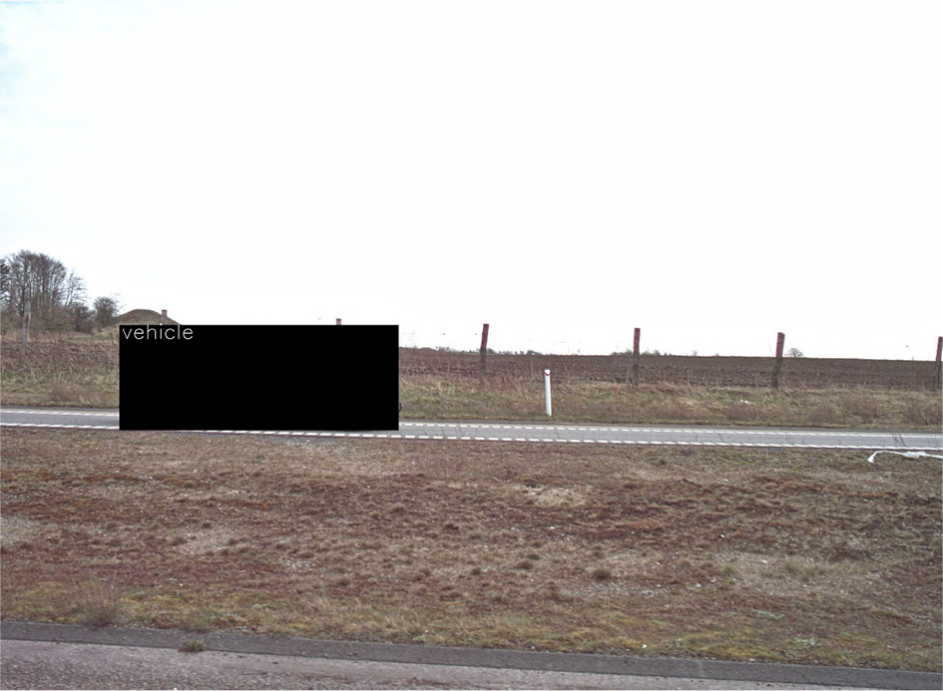
Automatic anonymization of car that appear in the image.

The following section contains sample images from the camera while driving. Figure 7 shows a sequence of three images that were acquired from a motorway near Aarhus in Denmark. The images were taken while the vehicle was traveling at 107 km/h and demonstrates a horizontal overlap of 61% at the start of the roadside. In the images, you can see that even objects that are close to the camera are sharp and it is possible to recognize the various plant species. The frame rate can also be set to match the driving speed so that the overlap between images is always 1/3 of the image width. Figure 8 shows an example of the automatic masking of vehicles.

## Conclusion

Invasive plant species present a pressing ecological challenge. Traditional monitoring methods have inherent limitations in coverage, timeliness and resources. This paper details the technical aspects and challenges of a high-speed, vehicle mountable camera system, that is designed for registering invasive plant species along roadways. So far, a total of 11 equivalent systems are currently being used and validated in 11 different countries. The data from such a camera system can provide an overview of the temporal and spatial distribution of invasive species, which can bring greater insight into how invasive plant species spread along roads. However, the data collected through a camera system like this possesses a dual purpose. Not only does it offer insights into invasive species dynamics, but it can also play a role in training advanced machine-learning algorithms, which could subsequently automate the time-consuming process of species recognition.

In combination with automated plant species recognition, this camera system paves the way for large scale cross country monitoring of invasive species spread.

## Data Availability

251 images from a test drive in July 2023 can be found in the following Zenodo repository: https://doi.org/10.5281/zenodo.10292599. All images are acquired when driving on highways with speeds >100 km/h. Data are available under the terms of the Creative Commons Attribution 4.0 International license (CC-BY 4.0).

## Software availability

Software for the web interface and for calculating motion blur is available in the following Zenodo repository: https://zenodo.org/doi/10.5281/zenodo.10287954.

Data are available under the terms of the Creative Commons Attribution 4.0 International license (CC-BY 4.0).
