# Supplementary material for: High-speed camera system for efficient monitoring of invasive plant species along roadways
Source: F1000Res. 2024 Oct 4;13:360. Originally published 2024 Apr 23. [Version 2] doi: 10.12688/f1000research.141992.2 (PMC11263905; doi:10.12688/f1000research.141992.2)
Supplement: Peer review history

# Reviewer report: Reviewer response for version 2

**DOI:** 10.5256/f1000research.172219.r329211

**Published:** 2024-10-16

**Authors:** Simona Ceschin, Lorenzo Pinzani

**Affiliations:** 1 Department of Science, Roma Tre University, Rome, Italy

In the revised version of this ms, the authors have made all the suggested modifications. Therefore, in my opinion this manuscript can be accepted.

 However, attention please that in the last sentence of the paragraph "Geotagging images" the word "planets" should be replaced with "plants".

Is the rationale for developing the new method (or application) clearly explained?

Yes

Is the description of the method technically sound?

Partly

Are the conclusions about the method and its performance adequately supported by the findings presented in the article?

Yes

If any results are presented, are all the source data underlying the results available to ensure full reproducibility?

Yes

Are sufficient details provided to allow replication of the method development and its use by others?

Partly

Reviewer Expertise:

Botany, Floristic Studies, Biodiversity Monitoring, Biological Invasion, R Statistical Package, Ecology and Evolution

We confirm that we have read this submission and believe that we have an appropriate level of expertise to confirm that it is of an acceptable scientific standard.

# Reviewer report: Reviewer response for version 2

**DOI:** 10.5256/f1000research.172219.r329210

**Published:** 2024-10-12

**Authors:** Lorenzo Palazzetti

**Affiliations:** 1 University of Florence, Florence, Italy

The authors have improved the manuscript according to reviewers' comments, so that, it can be accepted.

Is the rationale for developing the new method (or application) clearly explained?

Partly

Is the description of the method technically sound?

Yes

Are the conclusions about the method and its performance adequately supported by the findings presented in the article?

Yes

If any results are presented, are all the source data underlying the results available to ensure full reproducibility?

No source data required

Are sufficient details provided to allow replication of the method development and its use by others?

Yes

Reviewer Expertise:

Computer science

I confirm that I have read this submission and believe that I have an appropriate level of expertise to confirm that it is of an acceptable scientific standard.

# Reviewer report: Reviewer response for version 1

**DOI:** 10.5256/f1000research.155483.r300993

**Published:** 2024-07-22

**Authors:** Lorenzo Palazzetti

**Affiliations:** 1 University of Florence, Florence, Italy

The authors investigated the possibility of monitoring invasive plant species using a high-speed camera system mounted on the back of a vehicle.

 They provided a detailed description of the entire system, including its components and insights into the quality of the collected images.

 Additionally, they introduced a client-server service to store, anonymize, and geotag the images in real-time relying on an internet connection.

 I list below my main concerns:

1. The authors proposed a system for monitoring invasive plant species along roadways, claiming that it can address the issues of traditional monitoring systems, which are labor-intensive and highly dependent on human effort. While their system moves towards automated monitoring, their solution still requires a driver to traverse multiple roads to cover a large area, and a human operator must (at least) annotate the images after the ride to establish the basis for a machine learning model. Although the latter task can be shortened by leveraging the output of the remote controller, there remains a huge amount of data to process. Additionally, the proposed remote controller necessitates actions to be performed during the ride, making the task human-centered and time-consuming once again. Please discuss possible solutions to these challenges and clarify the role and usage of the remote controller in the system.
2. The authors introduced a remote controller for marking potential areas. From my understanding, this process is performed in real-time during a ride, and it may represent a potential issue for the accuracy of the entire system. The authors have to discuss the accuracy and the precision of this approach (i.e., how many marked locations were correct and how many were positive locations the operator missed) and eventually propose mitigation strategies.
3. The authors provided in Figure 2 an interesting analysis of the correlation between exposure time, speed, and perceived blurriness. Since perceived blurriness inside an image may vary according to the observer, the authors should provide more details on how they derived this indicator.
4. Since the system traces the movements of the vehicle on which the camera is mounted, potential privacy issues may arise. The authors have to discuss potential solutions to anonymize the vehicle's movements and protection against eavesdropping.

Is the rationale for developing the new method (or application) clearly explained?

Partly

Is the description of the method technically sound?

Yes

Are the conclusions about the method and its performance adequately supported by the findings presented in the article?

Yes

If any results are presented, are all the source data underlying the results available to ensure full reproducibility?

No source data required

Are sufficient details provided to allow replication of the method development and its use by others?

Yes

Reviewer Expertise:

Computer science

I confirm that I have read this submission and believe that I have an appropriate level of expertise to confirm that it is of an acceptable scientific standard, however I have significant reservations, as outlined above.

# Response

**Published:** 2024-09-19

**Authors:** Mads Dyrmann

**Affiliations:** The AI Lab ApS, Aarhus, Denmark

Thank you for your review. We have reviewed your comments and addressed them in an updated version of the article.

 Regarding your point 2, we do not have a measure for how many invasive plants an operator overlook and how timely they press the annotation button. To ensure that the invasive plants are actually recorded, we have built a buffer into the camera that ensures images are saved already one second before the button is pressed. However, while building the image library, we do not see it as critical if some invasive plants are overlooked and the button is not pressed, as long as these non-annotated images are not included in the dataset as examples of areas without invasive plants.

 Regarding point 3, we have considered blur under the assumption that the image is in focus and the lens is ideally sharp. Blur occurs when the camera is in motion during exposure. We report perceived pixel blur, as the blur occurs before the image is demosaiced, which reduces the observed blur through interpolation.

 I hope this clarifies our considerations when designing the system.

# Reviewer report: Reviewer response for version 1

**DOI:** 10.5256/f1000research.155483.r300995

**Published:** 2024-07-17

**Authors:** Daniel M Jenkins

**Affiliations:** 1 University of Hawaii, Manoa, Honolulu, USA

This manuscript provides a fairly straightforward technical description of a vehicle mounted camera system to record images of invasive plants along roadsides during travel. The work represents a significant amount of thought and engineering development to improve the efficiency for data collection, ensuring adequate resolution and speed to ensure complete coverage / overlap capturing a roadside scene. A key advance includes dynamic controls from a web interface, enabling a user to selectively capture specific scenes (reducing unnecessary storage, and effort for subsequent use in AI training or manual recording of target plant census / distribution), and automated control of shutter speed based on GNSS estimate of vehicle speed to minimize motion blur and ensure adequate frame rates. The considerations for design / selection of the optical components / characteristics is comprehensive and systematic. Interestingly the system includes an asynchronous AI engine to automatically “anonymize” people, vehicles, and bicycles recognized in images recorded into permanent external storage- this feels unnecessarily cumbersome for the intended application but an admirable feature to include nevertheless (it’s not clear to me whether privacy laws in Europe or elsewhere would require this for the intended application).

 It would be really helpful to publish at least some aspects of source code for the system, though I don’t assume that is a requirement for the journal and I understand this would make it more difficult to commercialize the technology for broader deployment (and I assume that is not a requirement for publication in this journal). Overall this writing is very succinct and represents a relatively comprehensive engineering description that would be very useful for the proposed application (I would be personally interested in using it in my own research), and the authors articulate sound reasoning for deploying the technology on a vehicle mounted system. I have only a handful of suggestions for improvement:

- Overall the writing is clear and concise, but there are a handful of issues with English grammar, notably in verb tenses in the second paragraph of the introduction.
- In the introduction, it would be helpful to include a citation, and / or specific examples of plants where the flowering status is critical for invasive plant ID  / differentiation (such that revisiting sites on a regular basis would be necessary for definitive identification)
- For justification of the specifications of the imaging sensor (description of the camera module), the authors use a systematic approach to ensure that resolution is adequate for identification of invasive plants, with global high speed shutter sufficient to prevent motion blur at the desired resolution (due to the moving vehicle). I think it would be helpful to refer more specifically to some of these design / operating constraints earlier in the manuscript (i.e. the tradeoffs between vehicle speed, shutter speed, and image blur in Figure 2, within the section on image sensor selection), though for the patient reader this probably isn’t critical.

Is the rationale for developing the new method (or application) clearly explained?

Yes

Is the description of the method technically sound?

Yes

Are the conclusions about the method and its performance adequately supported by the findings presented in the article?

Yes

If any results are presented, are all the source data underlying the results available to ensure full reproducibility?

Yes

Are sufficient details provided to allow replication of the method development and its use by others?

Partly

Reviewer Expertise:

Sensors and instrumentation

I confirm that I have read this submission and believe that I have an appropriate level of expertise to confirm that it is of an acceptable scientific standard.

# Response

**Published:** 2024-09-19

**Authors:** Mads Dyrmann

**Affiliations:** The AI Lab ApS, Aarhus, Denmark

Thank you very much for your review and interest in the system. We would like to capture the same species throughout a growing season to obtain the greatest possible variation within each species, which will contribute to the robustness of the AI models that are to be trained to recognize the species.

 For example, we would not expect a model to be able to recognize *Lupinus polyphyllus* in bloom if it has only been trained on wilted examples. This has been added to the introduction.

 We have also added a brief description of the size of objects and considerations regarding ground sampling distance to the section on "Recording, coverage and field of view considerations.

 I hope this clarifies our considerations when designing the system.

# Reviewer report: Reviewer response for version 1

**DOI:** 10.5256/f1000research.155483.r280723

**Published:** 2024-06-20

**Authors:** Simona Ceschin, Lorenzo Pinzani

**Affiliations:** 1 Department of Science, Roma Tre University, Rome, Italy

I suggest that the authors clarify on some technical aspects noted in the text of the Article.

 In particular: the inability for the camera to monitor both sides of the roadway at the same time; the distance at which the camera works best in relation to the width of the various European roads; the possible presence of a second operator to use remote control; The internal memory capabilities of the system.

 I suggest that the authors better expand the economic and technical limitations for the reproducibility of the method in other countries both in terms of the availability and cost of the instrumentation used and in terms of its applicability to different environmental and vegetation contexts (e.g. forests, areas with sparse or herbaceous vegetation).

 I personally found this article interesting and well balanced. I therefore only request some clarifications and minor modifications that could improve the understanding of the study.

 In general, the English language and punctuation should be revised to ensure that an international audience can clearly understand your text.

Is the rationale for developing the new method (or application) clearly explained?

Yes

Is the description of the method technically sound?

Partly

Are the conclusions about the method and its performance adequately supported by the findings presented in the article?

Yes

If any results are presented, are all the source data underlying the results available to ensure full reproducibility?

Yes

Are sufficient details provided to allow replication of the method development and its use by others?

Partly

Reviewer Expertise:

Botany, Floristic Studies, Biodiversity Monitoring, Biological Invasion, R Statistical Package, Ecology and Evolution

We confirm that we have read this submission and believe that we have an appropriate level of expertise to confirm that it is of an acceptable scientific standard, however we have significant reservations, as outlined above.

# Response

**Published:** 2024-09-19

**Authors:** Mads Dyrmann

**Affiliations:** The AI Lab ApS, Aarhus, Denmark

Thank you for your thorough review. We agree with your points and have therefore updated the article to address and clarify the issues.

 Regarding adding an extra camera module, it is possible from a technical perspective. It would however affect the development cost and fill the memory faster. Furthermore, annotation would become more cumbersome, as the passenger annotating with the remote control would have to monitor both sides of the road simultaneously.
